# MicroRNA-454 inhibits non-small cell lung cancer cells growth and metastasis via targeting signal transducer and activator of transcription-3

**DOI:** 10.3892/mmr.2022.12768

**Published:** 2022-06-09

**Authors:** Shuliang Liu, Xingping Ge, Lingfei Su, Aifeng Zhang, Xuri Mou

Mol Med Rep 17: 3979–3986, 2018; DOI: 10.3892/mmr.2017.8350

Following the publication of this paper, it was drawn to the Editors’ attention by a concerned reader that certain of the cell migration and invasion assay data shown in Figs. 2C and 4D were strikingly similar to data appearing in different form in other articles by different authors. Owing to the fact that the contentious data in the above article had already been published elsewhere, or were already under consideration for publication, prior to its submission to *Molecular Medicine Reports*, the Editor has decided that this paper should be retracted from the Journal. The authors were asked for an explanation to account for these concerns, but the Editorial Office did not receive a reply. The Editor apologizes to the readership for any inconvenience caused.

